# Pheromone and Host Plant Odor Detection in Eastern Spruce Budworm, *Choristoneura fumiferana* Clemens (Lepidoptera: Tortricidae)

**DOI:** 10.3390/insects14070653

**Published:** 2023-07-21

**Authors:** Thanusha Suresh, Lucas E. Roscoe, N. Kirk Hillier

**Affiliations:** 1 Biology Department, Acadia University, 33 Westwood Ave, Wolfville, NS B4P 2R6, Canada; thanushasuresh@acadiau.ca; 2 Canadian Forest Service-Atlantic Forestry Centre, 1350 Regent Street, P.O. Box 4000, Fredericton, NB E3B 5P7, Canada; lucas.roscoe@nrcan-rncan.gc.ca

**Keywords:** *Choristoneura fumiferana*, single sensillum recording, olfaction, mating disruption, olfactory receptor neuron

## Abstract

**Simple Summary:**

The spruce budworm (*Choristoneura fumiferana* Clemens) is a major defoliating pest of coniferous trees in North America. In recent decades, substantial advances in pheromone-mediated trapping and mating disruption technologies have provided researchers with renewed hope for novel population control strategies of *C. fumiferana*. While the chemical ecology of spruce budworm is continually being studied, a detailed study of the antennal sensilla in adults has yet to be completed. In this research, we review the state of knowledge of *C. fumiferana* chemical ecology and behavioral responses to chemical stimuli. Further, extracellular single sensillum recordings (SSR) were used to determine the response of olfactory receptor neurons (ORNs) in the antennal sensilla of male and female *C. fumiferana* to host plant volatiles, and female sex pheromones with a range of concentrations. Together, these data will improve knowledge of mechanisms by which adult *C. fumiferana* respond to pheromone and host plant volatiles and will provide insights that may improve development of integrated pest management strategies based on the chemical ecology of spruce budworm.

**Abstract:**

Spruce budworm, *Choristoneura fumiferana* Clemens, is an ecologically significant defoliator of spruce and balsam fir in North America. Optimization of semiochemical-mediated control is needed to improve the existing integrated pest management systems such as mating disruption and population estimation. This study used single sensillum recordings (SSR) to identify the responses of olfactory receptor neurons (ORNs) in the antennal sensilla of adult male and female *C. fumiferana* to host plant volatiles, and female sex pheromones. There have been few SSR studies done on spruce budworm, and to our knowledge, the present study represents the first attempt to examine the responses of ORNs from antennal sensilla in response to a range of host and conspecific stimuli. A total of 86 sensilla were characterized and sorted into 15 possible sensillum categories based on odor responses. We observed that specialist sensilla responding to few ligands were more abundant in both male and female than sensilla exhibiting more generalized odorant responses. (*E*/*Z*)-11-tetradecenal elicited responses from ORNs from any sensilla which were sensitive to pheromones in both males and females. Female *C. fumiferana* ORNs were able to detect and physiologically respond to female-produced sex pheromones with the same degree of sensitivity as their male counterparts. Together, these data improve our knowledge of mechanisms by which adult budworms respond to pheromone and host plant volatiles and provide insights that may be complementary to existing integrated pest management (IPM) strategies based on the chemical ecology of spruce budworm.

## 1. Introduction

Spruce budworm, *Choristoneura fumiferana* Clemens (Lepidoptera: Tortricidae), is an oligophagous eruptive forest defoliator in north-eastern North America [1,2]. It drastically impacts hundreds of thousands of hectares of economically and ecologically valuable fir and spruce [3]. Early-instar larvae feed preferentially on current year shoots of balsam fir (*Abies balsamea* (L.) Mill.), white spruce (*Picea glauca* (Moench) Voss), and black spruce (*Picea mariana* (Mill.) B.S.P.) [4,5]. Mature larvae feed on buds, flowers, and newly developing foliage of host trees, and can cause up to 87% defoliation in affected trees [6]. Dendrochronological evidence from ancient timbers suggests that outbreaks of *C. fumiferana* have a long history in Nova Scotia, New Brunswick, Newfoundland, Quebec, and Ontario [7]. More recently, in Quebec, the current budworm outbreak has damaged approximately 7.2 million hectares since in 2006 [8]. Given its significant impact, *C. fumiferana* represents a major source of ongoing and future defoliation in affected regions.

The detection and processing of volatile chemical signals is essential for the fulfillment of multiple purposes, including the location food, hosts, predators, oviposition sites, and potential mates [9,10,11,12]. Greenbank (1963) [13] demonstrated the importance of sex pheromone communication in *C. fumiferana*, with Weatherston et al. (1971) [14] subsequently documenting the primary sex pheromone as (*E*)-11-tetradecenal. This was further clarified as 96:4 (*E*/*Z*)-11-tetradecenal by Sanders and Weatherston (1976) [15], and the sex pheromone finally quantified as 95:5 (*E*/*Z*), with 3% (of E11-14:Ald) of a secondary component identified as (*Z*)-11-hexadecenal [16]. Pheromone traps baited with a synthetic blend of female sex pheromone blend have been used extensively to monitor *C. fumiferana* populations [17,18]. Pheromone-baited traps at the ground and tree canopy levels have been used to monitor *C. fumiferana* populations in Quebec from 2002 to 2012 with results showing that a conservative threshold of 100 males per trap at the ground level could be used as indicator to initiate significant forest management practices to control the transition from endemic to epidemic populations [19]. Recently in New Brunswick, dispersal of *C. fumiferana* using a citizen science protocol was developed and successfully used to track budworm population movement using traps baited with the synthetic sex pheromone blend [8]. Such monitoring has been essential to the direction of effective management operations in these regions.

In addition to fulfilling roles in population detection, pheromones can be a critical component of direct management strategies, such as pheromone-based mating disruption. Mating disruption involves the dissemination of synthetic sex-pheromones in an area to disrupt mate localization and/or courtship [20]. Mating disruption has been successfully implemented to control populations of several herbivorous insects including the oriental fruit moth, *Grapholita molesta* Busck (Lepidoptera: Tortricidae) [20,21,22,23], codling moth, *Cydia pomonella* L. (Lepidoptera: Tortricidae) [24,25], European grapevine moth, *Lobesia botrana* [Denis and Schiffermuller] (Lepidoptera: Tortricidae) [26] and light brown apple moth, *Epiphyas postvittana* (Walker) (Lepidoptera: Tortricidae) [27]. In 2007, Disrupt Micro-Flakes^®^ SBW (Hercon Environmental, Emigsville, PA, USA), was registered in Canada as a synthetic formulation of sex attractant for mating disruption of *C. fumiferana* [28]. Recently, mating disruption of *C. fumiferana* was initiated by aerial application of a registered formulation of synthetic spruce budworm female sex pheromone in 2008, 2013 and 2014 in Quebec. Though a 90% reduction in captures of male spruce budworm moths in pheromone-baited traps was observed, there was no reduced egg and larval density in the following generation [28]. Such divergent results between to moth capture and population densities indicate that further knowledge surrounding pheromone detection, and mechanisms of mating disruption is needed.

In addition to sex pheromones, host plant volatiles play an important role in orientation and dispersal within sites for lepidopteran insects [29,30,31]. For example, in *C. fumiferana*, choice of oviposition site is strongly influenced by volatiles emitted from the host needles [32,33]. Needle defense components are an important group of plant volatile those can influence oviposition in *C. fumiferana* [34]. Both field and laboratory experiments have demonstrated distinct patterns of terpenes in host plant that are perceived by different species of *Choristoneura* [13,35,36,37]. The less volatile constituents of host needles also influence the feeding response of *C. fumiferana* larvae [5]. Understanding how host plant volatiles are detected by *C. fumiferana* may therefore be useful in enhancing attractant technologies, such as pheromone lures and traps, and may further inform management plans.

Lepidoptera perceive volatiles, such as conspecific pheromones, by chemical activation of olfactory receptor neurons (ORNs) primarily housed within the antennal sensilla [38]. In many moth species, each sensillum will contain one to three ORNs that are sensitive to chemical cues [38]. Albert and Seabrook (1973) [39] documented the number and range of sensillum types present on the antennae of male *C. fumiferana*, (sensilla chaetica, sensilla trichodea, sensilla coeloconica, and sensilla styloconica). Overall, males had a higher number and density of sensilla relative to conspecific females [39]. Furthermore, electroantennogram responses showed that *C. fumiferana* females can autodetect pheromone components of their own species, with sensitivity exhibiting two-thirds the response amplitude of their male counterparts [40]. Albert et al. (1974) [41] recorded action potentials from antennae of male *C. fumiferana* to female sex pheromone, (*E*)-11-tetradecenal at the dosages from 10^−8^–10^−5^ mg, validating sensillar recording for this species.

The aim of this study was to determine the response profile of the antennae of *C. fumiferana* to an array of different behaviorally relevant olfactory cues, including female sex pheromones and host plant volatiles in both sexes through usage of single sensillum recording (SSR) technique. Specifically, ORN spike frequencies and amplitudes were evaluated against increasing dosages of behaviorally relevant stimuli to determine relative antennal sensitivity to these cues. Previous work has shown that sexual dimorphism is present on antennae of *C. fumiferana*, as well as in male and female electroantennogram responses. Ergo, it was predicted that ORNs responses from males and females would differ significantly. Lastly, we predicted higher overall sensitivity to the major sex pheromone component, (*E*/*Z*)-11-tetradecenal (95:5), in comparison to other pheromone components: (*Z*)-11-tetradecenal and (*Z*)-11-hexadecenal.

## 2. Materials and Methods

### 2.1. Insects

Pupae of *C. fumiferana* were acquired from the Insect Production and Quarantine Laboratory, Great Lake Forestry Centre (Sault Ste. Marie, ON, Canada). Male and female pupae were separated into different containers and reared in an environmentally controlled insectary room (24 °C, 60% relative humidity, and a reverse light cycle [14D:10L]). Newly emerged adults were removed from rearing containers and stored in the same environmentally controlled insectary room. Three-to-seven-day-old adult males and females of *C. fumiferana* from the colony were used for electrophysiology.

### 2.2. Chemical Stimuli

Chemicals were selected based upon behavioral and biological relevance shown from previous studies (Table 1). Odorants included the primary female sex pheromone blend (hereafter ‘major component’)_ and two known female sex pheromone components: 95:5 (*E*/*Z*)-11-tetradecenal, (*Z*)-11-tetradecenal, (*Z*)-11-hexadecanal [15,17,42,43,44]; and nineteen host plant volatiles induced from larval feeding; (+) limonene, (+)-3-carene, alpha-humulene, (+)-alpha-pinene, beta-caryophyllene, camphene, farnesene (mix), jasmonic acid, linalool, myrcene, terpinolene, hexyl acetate, (*E*)-2-hexen-1-ol, (*Z*)-3-hexenol, hexanal, (+/−) camphor, (*E*)-2-hexen-1-al, 1-hexanol and (-) bornyl acetate [45,46,47].

One molar stock solutions were made for each selected odorants in hexane, followed by dilution into decadic steps to a series of solutions ranging from 0.01 μg/μL to 10 μg/μL. To prepare stimulus cartridges, filter papers were cut into (35 mm × 7 mm) strips, and then fitted inside Pasteur pipettes. A total of 10 μL of a given dilution was applied using a microsyringe on a filter paper strip to provide a total load delivered ranging from 100 ng to 100 μg. A hexane blank was prepared by pipetting 10 μL of hexane onto filter paper. Loaded strips were allowed to evaporate for 20 s in a fume hood and then Pasteur pipettes were air sealed and wrapped in aluminum foil. Pipettes were kept in the freezer between experiments and brought to room temperature again before use. Cartridges were replaced with fresh stimuli after twenty puffs (or every two weeks).

### 2.3. Single Sensillum Recording (SSR)

Methods for single sensillum recording were similar to those used by Hillier et al. (2006) [31], Olsson and Hansson (2013) [48], and O’Connell [49]. Briefly, the insect preparation was mounted on a Nikon Eclipse Fixed Stage Microscope (Nikon; Mississauga, ON, Canada) and viewed under 300× magnification. Three-to-seven-day-old adult male and female budworms were restrained into a 10 μL pipette tip (with both ends cut to accommodate and hold the insect). A tungsten reference electrode was then inserted into the eye on the opposite side. A continuous flow of charcoal-filtered, humidified air was provided at a flow rate of 1 l/min from a glass tube 1.5 cm from the head of the moth, pointing at the antenna. A stimulus controller (Syntech CS-55; Ockenfels Syntech, Buchenbach, Germany) was used to switch the airstream from the continuous flow to the stimulus cartridge controlled by Autospike32 software (Ockenfels Syntech, Buchenbach, Germany). Both the stimulus and continuous flow were connected to a glass tube that functioned as a mixing chamber for clean humidified air and stimulus-laden air. The exit of the mixing chamber was positioned 10 mm away from the insect antenna.

An electrolytically sharpened tungsten microelectrode slowly lowered to the base of randomly selected trichoid sensilla using a motorized model micromanipulator (Ockenfels Syntech, Buchenbach, Germany) located in the first ten segments of the antennae from the proximal ventral surface (the antenna is 46 segments long in males, 45 segments long in females [39]). Trichoid sensilla were morphologically identified through comparison with results of a previous study on morphology and histology of the antenna of the male eastern spruce budworm, *C. fumiferana* [39]. When a sensillum was contacted, an initial response screening to each and all stimuli at 10 μg for a 100 ms pulse was conducted. Stimuli were presented in random order, with 1 min intervals between stimuli to prevent adaptation. A hexane blank was tested in between all stimulus presentations. If an ORN within a sensillum responded to a particular stimulus (indicated by changes in spike frequencies), all four doses (100 ng, 1 μg, 10 μg, 100 μg) were then screened. A maximum of 3 sensilla were tested per individual.

Signals were amplified via a Syntech universal AC/DC probe (Ockenfels Syntech, Buchenbach, Germany) with a gain of 10×. Signals were acquired with an IDAC-4 controller (Ockenfels Syntech, Buchenbach, Germany), inputted to a computer via a 16-bit analog-digital converter, and analyzed off-line with using AutoSpike32 software. The low cutoff filter setting was 50 Hz, and the high cutoff was 5 kHz. Changes in spike frequency following stimulation were recorded by counting the number of spikes during 500 ms following each stimulus onset and standardized by subtracting the number of spikes during the 500 ms of pre-stimulus recording time. Spike frequencies observed from stimulation with a hexane blank were subtracted from corresponding treatment stimuli to obtain the net change in spike frequency due to a given stimulus.

All statistical data were analyzed using R version 4.1.1, coupled with R-Studio, and using packages tidyverse, plotrix, stats, agricolae, and ggplot2. Main and interaction effects four-way ANOVA (Table 2) was used to determine significant differences in aggregatespike frequency based on odorant, dosage, sex and amplitudes (indicating different co-localized neurons), and means were separated using Fisher’s LSD test (p < 0.05). Original spike trains were colored by using vector graphics software, Inkscape (Inkscape’s Contributors and The Inkscape Project).

## 3. Results

In total, good electrical contacts (stable, observable spikes) were made in ORNs from 582 sensilla—58% males (338 sensilla) and 42% females (244 sensilla). A total of 22 components, including 3 female sex pheromones, and 19 host plant volatiles were screened against these sensilla to determine their range of responses. Most sensilla tested housed two physiologically active and odorant-responsive ORNs. In total, data were collected from 109 sensilla (61 males, 48 females) housing 218 ORNs (138 males, 80 females) that responded to at least one of the components at the 10 µg dosage. In total, 86 sensilla (55 from males, 31 from females, Table 3) contained 154 ORNs (98 from males, 56 from females) that had connections strong enough to permit analysis of a full complement of odorants and dosages (increasing dosages from 100 ng to 100 µg). ORNs were recorded from another 23 sensilla (12 males, 11 females) with incomplete recordings in which contact was lost prior to completing testing with the entire odorant series.

In males, at least one or more ORNs were found that responded to some combination of all odorants screened when stimulated at the 10 µg dose (Table 3). In females, twenty-one out of twenty-two odorants tested elicited responses from ORNs in one or more sensillum types at the 10 µg dose. In females, (-) bornyl acetate was the only component that did not elicit responses in any of the sensilla contacted. Based on the response profiles of ORNs housed in sensilla of both males and females, together 15 ‘sensillum types’ were identified (Table 3), based upon shared responses to the combination of stimuli.

Responses were also recorded with increasing dosage of odorants. Successful recordings were obtained following stimulation with 19 out of 22 odorants in males and 14 out of 22 in females. Significant differences were not noted when comparing between the sexes of *C. fumiferana* (Table 2, Main effects ANOVA, *Fisher’s LSD test*, *p* < 0.05).

To investigate the effect of stimulus dosage on the neuronal responses of ORNs to the range of odorants, ORN responses in the sensillum to different doses were tested. A total of 40 recordings were made with each odorant (each dosage with 10 recordings: 10 + 10 + 10 + 10). In this methodology, different spike amplitudes (high spike and low spike) were also distinguished in a single active ORN.

### 3.1. Responses to Female Sex Pheromones

**Figure 1:**
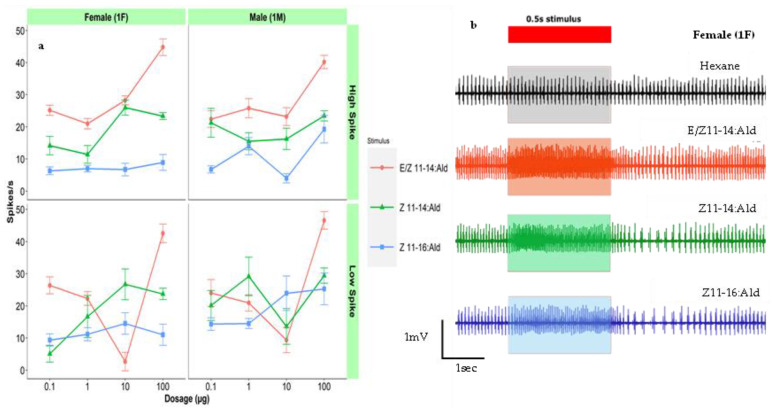
Olfactory Receptor Neurons (ORN) responses to female sex pheromones in Choristoneura fumiferana. (**a**) Dose–response curves measured in mean spikes/s above hexane blank (+-se) from ORNs in sensillum type 1 (1M, 1F: *n* = 10) responded to (E/Z)-11-14:Ald, (Z)-11-14:Ald and (Z)-11-16:Ald. (**b**) ORN response (original spike trains) from female; sensillum type 1 responded to (E/Z)-11-14:Ald, (Z)-11-14:Ald and (Z)-11-16:Ald at 100 µg.

Both major and minor component specialist pheromone cells were found in the same senillum in male and female *C. fumiferana.* In categorized sensillar types, Sensillar type 2 only responded to (*E*/*Z*)-11-tetradecenal (Table 3). However, in the sensillum type 1, both of the ‘a’ and ‘b’ neurons responded to either (*E*) or (*Z*) isomers (Figure 1, Table 3). For three sex pheromones, both sexes showed statistically significantly dose-dependent responses to primary sex pheromone component: (*E*/*Z*)-11-tetradecenal (Figure 1 and Figure S1a,b). This was also true between the dosages from 100 ng to 100 µg (Figure 1 and Figure S1a,b). At the highest dosage, all pheromones were significantly different from each other in both sexes, and (*E*/*Z*)-11-tetradecenal showed the strongest response (Figure S2a,b). The same pattern of responses was noted in all three female sex pheromones in male and female *C. fumiferana* (Figure S3). The frequency of spikes elicited in both cells were higher in (*E*/*Z*)-11-tetradecenal at the 100 µg dosage than other three dosages (Figure S3). (*Z*)-11-hexadecenal produced significantly lower responses in females than (*E*/*Z*)-11-tetradecenal or (*Z*)-11-tetradecenal in both spike amplitudes (Figure S2a,b).

### 3.2. Responses to Host Plant Volatiles

**Figure 2:**
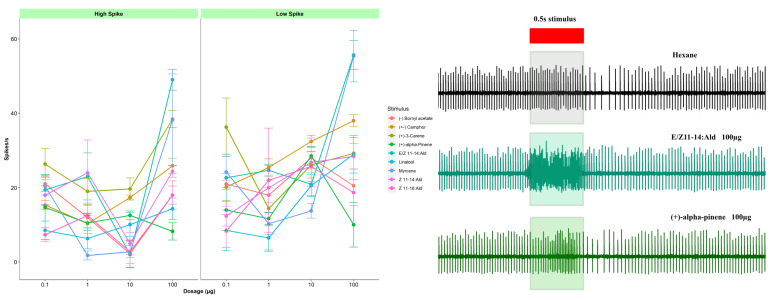
Olfactory receptor neurons (ORN) responses to female sex pheromones and host plant volatiles in Choristoneura fumiferana. (**a**) Dose–response curves measured in mean spikes/s above hexane blank (+-se) from ORNs in sensillum type 13 (13M) in male responded to (E/Z)-11-14:Ald, (Z)-11-14:Ald, (Z)-11-16:Ald, (−) bornyl acetate, (+/−) camphor, (+)-3-carene, (+)-alpha-pinene, linalool and myrcene. (**b**) ORN response (original spike trains) from male; sensillum type 13 responded to (E/Z)-11-14:Ald and (+)-alpha-pinene at 100 µg.

Data for each sex, stimulus, dosage, and spike amplitude were analyzed by multi-way ANOVA and post hoc *Fisher’s LSD tests* (*p* < 0.05). Dose–response testing showed similar patterns for both sexes for individual odorants (Figure 2 and Figure S4a,b). Following host plant volatile stimulation, individual spikes from different neurons could be reliably separated. Overall amplitude differences between neurons also were generally distinguishable. The majority of ORNs sensillum responses to host plant volatiles were dose-dependent with greater increases in firing frequency observed at higher doses of odorant (Figure S4a,b). Camphene elicited significantly higher neuron firing in low and high spike amplitude neurons recorded from females at 100 µg (Figure S4b: low spike amplitude 40 spikes/s, high spike amplitude > 40 spikes/s). In males, significantly higher responses were observed in myrcene at higher dosage (Figure S5a: low and high spike amplitudes > 40 spikes/s). (+)-alpha-pinene and farnesene (mix) elicited higher responses at the lower dosages in females (Figure S5b). However, in males, the responses were similar throughout the series of dosage (Figure S4a). Most of the host plant volatiles were significantly differed from each other in both females and males (Figure S5a,b). There were significant differences noted in between the stimulus load (100 ng–100 µg) in both male and female (Figure S5a,b). In many ORNs, significant differences were not noted between the responses to selected odorants by low and high spike amplitude ORNs; in females: (+)-3-carene, (+)-alpha-pinene, linalool, myrcene, and terpinolene, in males: (+)-alpha-pinene, beta-caryophyllene, camphene, linalool, myrcene, and terpinolene (Figure S6). The remaining five host plant volatiles ((+/−) camphor, (*E*)-2-hexen-1-ol, (*Z*)-3-hexen-1-ol, hexanal, and bornyl acetate) were analyzed separately as those did not have sufficient recordings from females for analysis.

In comparison to (*Z*)-3-hexen-1-ol, hexanal, and bornyl acetate, significantly higher firing frequencies were observed in (+/−) camphor and (*E*)-2-hexen-1-ol at 100 µg for high spike amplitude ORN (Figure S7). To hexanal, ORNs elicited significantly higher responses at the 1 µg dosage for high and low spikes amplitude with the firing frequency of >30 spikes/s (Figure S7). Comparatively, aggregate responses from low spike amplitude ORNs showed higher significant differences between the stimulus loading than high spike amplitude (Figure S8). In this five-stimulus panel in males, higher responses were observed in (*Z*)-3-hexenol, and hexanal (Figure S8). Aggregate frequencies of low spike amplitude ORNs observed were higher than high-spiking ORNs in response to more of the stimuli: (+/−) camphor, (*E*)-2-hexen-1-ol, (*Z*)-3-hexen-1-ol, and hexanal (Figure S9).

## 4. Discussion

This research has documented odor-induced responses from olfactory receptor neurons housed within trichoid sensilla on the antennal surface of *C. fumiferana*. Nearly 600 sensilla in male and female were contacted and tested against a panel of behaviorally relevant odorants. Responses to at least one of the odorants from the test panel were noted in 109 sensilla (housing 218 ORNs).

In total, 60% of sensilla tested contained ORNs which elicited responses to at least one of the pheromones (65% from male, 52% from female). (*E*/*Z*)-11-tetradecenal elicited responses in all sensilla which housed ORNs sensitive to any of the pheromones tested (Table 3). (*E*/*Z*)-11-tetradecenal was identified as a primary sex pheromone component in *C. fumiferana* many years ago [15,17,44] and ORNs sensitive to this component were noted in abundance from males (*n* = 36) and females (*n* = 16). Our results substantiate previous work [41,50,51] that report male *C. fumiferana* have sex-pheromone receptive ORNs in long sensilla trichodea.

In terms of the neurons responding selectively to the major and minor components, it is possible that the accuracy in discerning these responses is confounded by the 5% Z11-14:Ald impurity in the E11-14:Ald stimulus. For example, the male low spiking ‘2a’ ORN selectively responded only to the 95% E11-14:Ald/5% Z11-14:Ald mix, but not to Z11-14:Ald on its own (Table 3). Conversely low and high spiking ORNs in male type 1 and type 3 sensilla responded to both pheromone component stimuli. This may suggest that the non-specific response seen here is an artefact of the impurity, in which case, both low spiking (E11-14:Ald), and high spiking (Z11-14:Ald) ORNs, were activated by the presence of different ligands present in the mixture. Based on the data presented in Table 3 (and the assumption that the impurity is impacting results), both male and female *C. fumiferana* may exhibit both colocalized specialist ORNs (such as seen in housed in sensillum type 1 in both males and females), as well as sensilla which only house ORNs responding to E11-14:Ald (type 2). The former sensillum type (type 1) being the most abundant on the antenna.

**Figure 3:**
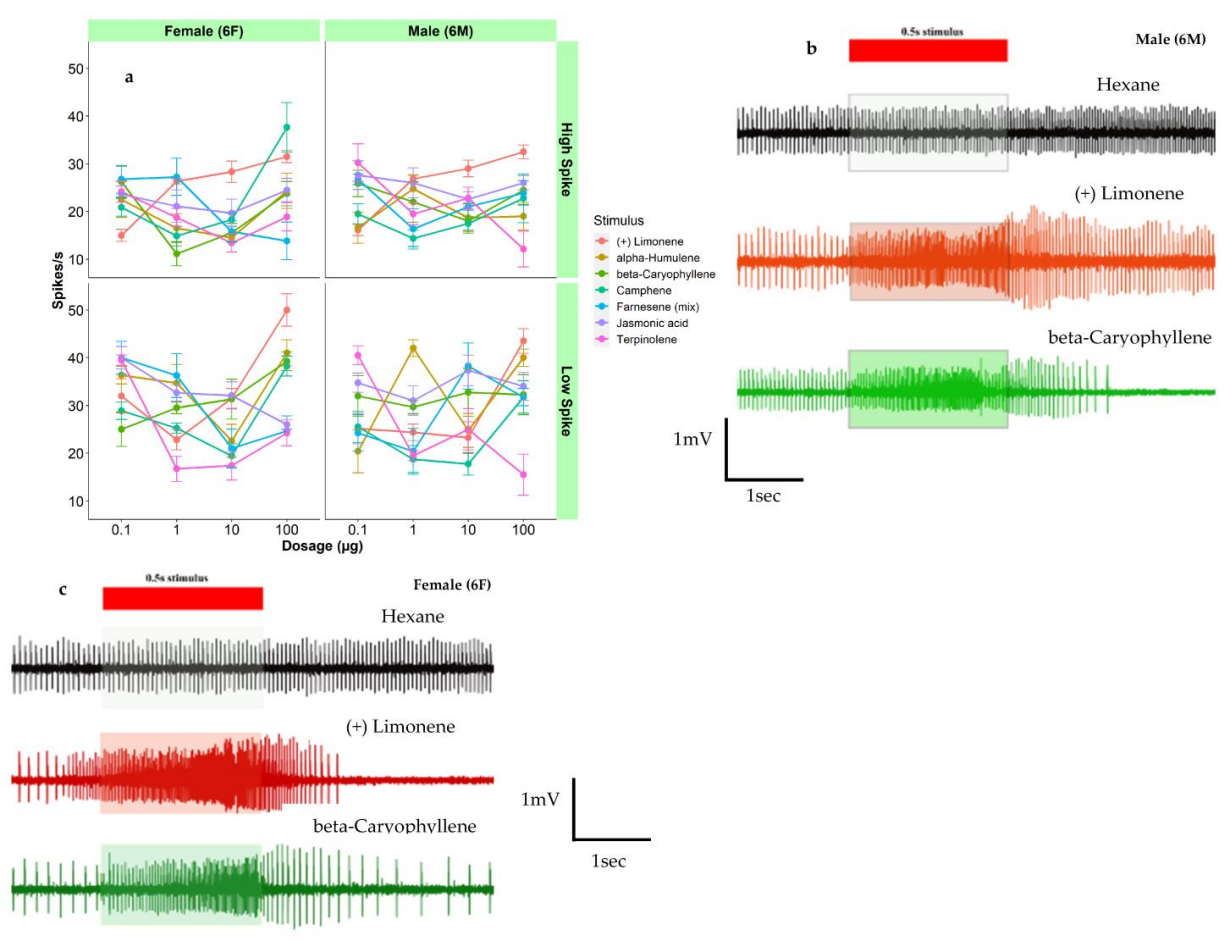
Olfactory receptor neurons (ORN) responses to host plant volatiles in Choristoneura fumiferana. (**a**) Dose–response curves measured in mean spikes/s above hexane blank (+-se) from ORNs in sensillum type 6 (6M, 6F) responded to (+) limonene, alpha-humulene, beta-caryophyllene, camphene, farnesene (mix), jasmonic acid and terpinolene. ORN response (original spike trains) from male (**b**) and female (**c**); sensillum type 6 responded to (+) limonene and beta-caryophyllene at 10 µg.

The majority sensilla contacted had one large spike amplitude ORN and one small spike amplitude ORN and both ORNs responded to female sex pheromones in a very similar way. This phenomenon was observed common in other moth species [38]. Most of our does response curves showed a consistent drop in the 3rd dose (10 μg) (Figure 1a, Figure 2a and Figure 3a). This may be due to insufficient peristimulus time. Future studies may be needed to find out the optimal peristimulus time in between the stimulus puff in *C. fumiferana.* As well, many responses did not exhibit ‘typical’ sigmoid response curves to stimuli tested, which may indicate that dosages were relatively high or low relative to individual ORN thresholds to response.

### 4.1. Sensitivity to Female Sex Pheromones

Ninety five percent purity (*E*/*Z*)-11-tetradecenal was used to test the responses of ORNs and this may also have influences in the results for (*Z*)-11-tetradecenal. Since the response of olfactory receptor neurons to key ligands and blends thereof are not additive, it is possible that the impurity of the 5% Z-isomer may have effected ORN responses to this blended stimulus [49]. Future studies should endeavor to use higher purity E-tetradecenal as a stimulus. Several studies have investigated detection and courtship behaviors of male *C. fumiferana* to sex pheromones released by female [42,50,51,52].

There were good dose-dependent trends for stimulus dosage and ORN firing frequency in the first 500 ms following stimulus presentation (Figure 1b and Figure 2b), manifested as an excitatory phase followed by an inhibitory phase. The same phenomena were observed in the male moth *Agrotis ipsilon* Hufnagel [53]. Under similar experimental conditions, *C. fumiferana* showed dosage-dependent responses to pheromone for 400 msec after stimulation [41].

Based on low and high spike amplitudes, our results indicated that, in all sensilla, two differentially tuned ORNs were responding to the different combinations of stimuli (Table 3, Figure 1). However, it is possible that two differently tuned ORNs co-compartmentalized within the same sensillum can exhibit same impulse amplitudes [54]. In the tortricid moth, *C. pomonella*, cross-adaption studies of the codlemone type receptor showed a reduced response to all codlemone isomers and *E*8, *E*10-12:Ac a short time (5 s) after stimulation with *E*8,*E*10-12:OH at a high dose of 10 µg and less adaptation when stimulated with the same dose of *E*8,*Z*10-12:OH [55]. Another study on *Heliothis subflexa* Guenée (Lepidoptera: Noctuidae) and *Heliothis virescens* F. (Lepidoptera: Noctuidae) to components of their sex pheromones confirmed the presence of two ORNs usually having different spike sizes and tuned to different components ((*Z*)-11-16Ac and (*Z*)-11-16:OH) [56]. However, the same study showed that in *H. subflexa*, (*Z*)-11-16Ac tuned neuron also elicited responses to (*Z*)-9-14Ald with nearly equivalent sensitivity [57]. Future work on *C. fumiferana* should integrate SSR cross-adaptation studies to discretely determine how many ORNs may be contributing to the spike trains from a given stimulus.

In *G. molesta*, recordings from projection neurons stimulated by pheromone components and plant volatiles showed different response patterns, whereas the dose-dependent responses differed between neurons and tested chemicals [58]. Likewise in budworm’s dose-dependency also showed highly variable neuronal activity between and within ORNs with both low and high spike amplitudes (Figures S1a,b, S4a,b and S7). However, in most of the ORNs recorded, spike frequency increased with higher doses; in males—58%, in females—64%, showing that the level of saturation was not reached (Figures S1, S4 and S7). In some moth species, such as *S. littoralis*, males exhibit much higher sensitivity to female sex pheromones [58]. However, in contrast to this, in *C. fumiferana*, males and females have similar ORN sensitivity to female sex pheromones tested. This is similar to previous observations of intracellular antennal lobe projection interneuron sensitivity from *G. molesta* [59].

Studies [51,60] have concluded that both female and male *C. fumiferana* have dose-related behavioral responses to the primary female sex pheromone blend. Walking, flexing of the body without the extrusion of the ovipositor, extrusion of the ovipositor with or without body flexion, ovipositional behavior, and antennal grooming were all dose-dependent in frequency of observation following exposure to female sex pheromone. At high dosages, levels of response were independent from dosage, presumably due to adaptation or habituation of the sensory system of *C. fumiferana*. Relative volatilities of each tested odorants must be considered when comparing ORNs responses, as vapor pressures of the individual odorant molecules could have the different emission rates, despite being loaded at the same dosage [61,62]. Amounts of impurities in test stimuli and unspecific responses associated with high dosage may also obscure true ligand-receptor relationships [62,63,64]. Research on *G. molesta* concluded that females did not respond to female-produced sex pheromones and furthermore suggest that female autodetection observed in other species may be largely artefacts of high dosages or impurities in stimuli [56]. However, in this study most of the odorants selected had relatively similar size, vapor pressures and high purity. Future behavioral work investigating if females demonstrate distinct behavioral responses to conspecific blends versus generic sensitivity to selected components would clarify if the responses observed are autodetection sensu stricto [65].

### 4.2. Female Pheromone Autodetection

Previous work has shown that female *C. fumiferana* can perceive their own pheromone and become more active in pheromone permeated air [51,52,66]. Our results reveal that female *C. fumiferana* can detect, and physiologically respond to female-produced sex pheromones with the same degree of sensitivity as their male counterparts (Figure S1a,b). This is similar to observations made by intracellular recordings [58] from *G. molesta*, in which adults of both sexes are able to detect and respond to the pheromone emitted by the females.

Recent studies have investigated the significance of female autodetection, which is detection of a pheromone by an individual producing that component. Autodetection may lead to internal physiological changes that cannot be observed behaviorally and could impact hormonal activity, mating receptivity, rate of pheromone synthesis, or induce dispersal in conspecific females [65]. Dispersal behavior by clustered females has been documented in *C. fumiferana*, previously [51,66]. Both *G. molesta* and *Choristoneura rosaceana* (Harris) (Lepidoptera: Tortricidae) also engage in increased movement [67] following pheromone pre-exposure. Olfactometer tests also showed that females of the noctuid moths *H. armigera* and *H. zea* are repelled when presented with their own pheromone [68]. In *S. littoralis*, females have reduced mating and increased flight activity following pheromone exposure [69], whereas females of some other species, *Vitacea polistiformis* Harris (Lepidoptera: Sesiidae), and *Ephestia kuehniella* Zeller (Lepidoptera: Pyralidae), were repelled and exhibited increased local movements [70]. Increased flight activity and local movement will increase chances of mating of unmated females and reduce post-mating competition for oviposition site, which would otherwise be detrimental to progeny due to lack of food [65,71,72].

### 4.3. Sensitivity to Host Volatiles

Response to the host plant volatile (+)-α-pinene were frequently co-localized with other host plant volatiles: myrcene, linalool, α-humulene, (+)-3-carene, (-) bornyl acetate, and (+) camphor and sex pheromones (Figure 2). The green leaf volatile (*E*)-2-hexenal also produced consistent responses by males and females when presented at a 10 µg load (Table 3). Strong responses to (*E*)-2-hexenal and linalool observed in the current study have also been found in the tortricid grapevine moth *L. botrana* [73].

Blends of pheromone and host plant volatiles encountered under environmental conditions may further influence nature of responses by ORNs profiled in this study. A synergetic effect of green leaf volatiles blends on the responses to sex pheromone has been observed in males of *G. molesta* [74,75]. In males of noctuid moth, *A. ipsilon*, plant volatiles heptanal [76,77] and linalool [78] were found to reduce pheromone sensitivity at the peripheral and central olfactory level. In *H. zea*, linalool and (*Z*)-3-hexen-1-ol were found to increase the response of pheromone specific ORNs when presented simultaneously with the main pheromone component [79]. Furthermore, dosage-dependent mixture suppression was identified pheromone and plant volatiles in *H. virescens* [80]. Future work, using in vivo calcium imaging in the antennal lobe, intracellular recordings of neurons in the macroglomerular complex, or wind tunnel experiments could illuminate synergetic and additive effects of such components in *C. fumiferana* as well [81].

This study provides insight regarding the organization and sensitivity of olfactory receptor neurons within antennal sensilla of *C. fumiferana*. Our results provide a basis for which compounds mediate host attraction and which may act as synergists for pheromones due to co-localization.

## Figures and Tables

**Table 1:** Chemical stimuli used for single sensillum recording.

Stimulus Component	Purity (%)	CAS Number	Supplier
Female sex pheromones			
(*E*)-11-14:Ald *	95%	Mixture	Bedoukian
(*Z*)-11-14:Ald	95%	35237-64-0	Bedoukian
(*Z*)-11-16:Ald	95%	53939-28-9	Bedoukian
Host plant volatiles			
(+)-α-Pinene	98%	7785-70-8	Aldrich
Myrcene	90%	123-35-3	Aldrich
Linalool	97%	78-70-6	Aldrich
(+)-3-Carene	92%	13466-78-9	Sigma-Aldrich
(-) Bornyl acetate	95%	5655-61-8	Sigma-Aldrich
(+/−) Camphor	95%	76-22-2	Sigma-Aldrich
α-Humulene	95%	6753-98-6	Aldrich
β-Caryophyllene	80%	87-44-5	Sigma-Aldrich
Farnesene (mix)	67%	502-61-4	SAFC
Camphene	80%	456055	Sigma-Aldrich
(+) Limonene	96%	5989-27-5	Fluka
Terpinolene	87%	586-62-9	Fluka
Jasmonic acid	95%	77026-92-7	Sigma-Aldrich
Hexanal	98%	66-25-1	Aldrich
1-Hexanol	95%	111-27-3	Sigma-Aldrich
Hexyl acetate	99%	142-92-7	Aldrich
(*E*)-2-Hexen-1-al	98%	6728-26-3	SAFC
(*Z*)-3-Hexen-1-ol	99%	928-96-1	Bedoukian
(*E*)-2-Hexen-1-ol	95%	928-95-0	SAFC

* <5% (Z)-11-14:Ald is present as a contaminant. Highest purity available.

**Table 2:** Main and interaction effect of analysis of variance (ANOVA) for stimulus, dosage sex and amplitude.

		Df	Sum Sq	Mean Sq	F Value	Pr(>F)
Stimulus		18	54,099.35	3005.51	35.45	<0.001
Dosage		3	24,991.08	8330.36	98.25	<0.001
Sex		1	2.92	2.92	0.03	0.85
Amplitude		1	13,478.6	13,478.6	158.98	<0.001
Stimulus:Dosage		54	46,651.62	863.91	10.19	<0.001
Stimulus:Sex		13	11,438.8	879.90	10.37	<0.001
Dosage:Sex		3	774.15	258.05	3.04	0.02
Stimulus:Amplitude		18	4955.15	275.28	3.24	<0.001
Dosage:Amplitude		3	329.23	109.74	1.29	0.27
Sex:Amplitude		1	395.59	395.59	4.66	0.03
Stimulus:Dosage:Sex		39	19,030.92	487.97	5.75	<0.001
Stimulus:Dosage:Amplitude		54	7472.84	138.38	1.63	<0.01
Stimulus:Sex:Amplitude		13	3138.16	241.39	2.84	<0.001
Dosage:Sex:Amplitude		3	1204.22	401.40	4.73	<0.01
Stimulus:Dosage:Sex:Amplitude		39	6712.72	172.12	2.03	<0.001
Residuals		1624	13,7681.1	84.77		

Df—Degrees of freedom; Sum Sq—Sum of the Square of variation; Mean Sq—Mean Square of variation; F value—value on the F distribution; Pr—Probability.

**Table 3:** Fifteen olfactory receptor neuron response profiles in both male and female *Choristoneura fumiferana* at a 10 µg dosage with two different spike amplitudes.

Sensillar Category	Female Sex Pheromones												Host Plant Volatiles																				Broadly Tuned							
Sensillar Type	1				2				3				4		5		6				7		8				9		10		11		12		13		14		15	
Sex	Male		Female		Male		Female		Male		Female		Female		Male		Male		Female		Male		Male		Female		Male		Male		Male		Male		Male		Male		Male	
	1a	1b	1a	1b	2a	2b	2a	2b	3a	3b	3a	3b	4a	4b	5a	5b	6a	6b	6a	6b	7a	7b	8a	8b	8a	8b	9a	9b	10a	10b	11a	11b	12a	12b	13a	13b	14a	14b	15a	15b
Female sex pheromones																																								
*E*/*Z*11-14:Ald	+	++	0	+++	++	0	+++	++	++	0	++	0	0	0	0	0	0	0	0	0	0	0	0	0	0	0	0	0	0	0	0	0	+	0	++	0	+	0	0	+
*Z*11-14:Ald	+	++	+	+++	0	0	0	0	++	0	+	0	0	0	0	0	0	0	0	0	0	0	0	0	0	0	0	0	0	0	0	0	+	0	+++	+	0	0	0	+
*Z*11-16:Ald	++	+	+++	+	0	0	0	0	0	0	0	0	0	0	0	0	0	0	0	0	0	0	0	0	0	0	0	0	0	0	0	0	++	0	+++	0	0	0	++	+
Host plant volatiles																																								
(+)-α-Pinene	0	0	0	0	0	0	0	0	0	0	0	0	++	++	+	0	0	0	0	0	0	0	0	0	0	0	0	0	0	0	0	0	+	+	+++	+	+	0	0	+
α-Humulene	0	0	0	0	0	0	0	0	0	0	0	0	0	0	0	0	+++	++	++	++	0	0	0	0	0	0	0	0	0	0	0	0	0	0	0	0	0	0	0	0
β-Caryophyllene	0	0	0	0	0	0	0	0	0	0	0	0	0	0	0	0	+++	++	+++	++	0	0	0	0	0	0	0	0	0	0	0	0	0	0	0	0	0	0	0	0
Myrcene	0	0	0	0	0	0	0	0	0	0	0	0	+	+	0	0	0	0	0	0	0	0	0	0	0	0	0	0	0	0	+	0	0	0	++	0	+	0	+	++
Linalool	0	0	0	0	0	0	0	0	0	0	0	0	++	++	0	0	0	0	0	0	0	0	0	0	0	0	0	0	0	0	+	0	0	0	++	+	+	0	0	0
Jasmonic acid	0	0	0	0	0	0	0	0	0	0	0	0	0	0	0	0	+++	++	+++	++	0	0	0	0	0	0	0	0	0	0	0	0	0	0	0	0	0	0	0	0
(+)-3-Carene	0	0	0	0	0	0	0	0	0	0	0	0	++	++	0	0	0	0	0	0	0	0	0	0	0	0	0	0	0	0	0	0	0	0	+++	++	+	0	0	0
Farnesene (mix)	0	0	0	0	0	0	0	0	0	0	0	0	0	0	0	0	+++	++	++	++	0	0	0	0	0	0	0	0	0	0	0	0	0	0	0	0	0	0	0	0
(-) Bornyl acetate	0	0	0	0	0	0	0	0	0	0	0	0	0	0	+	0	0	0	0	0	0	0	0	0	0	0	0	0	0	0	+	0	0	++	+++	0	+	0	0	0
(+) Limonene	0	0	0	0	0	0	0	0	0	0	0	0	0	0	0	0	++	+++	+++	+++	+	+++	0	0	0	0	0	0	0	0	0	0	0	0	0	0	0	0	0	0
(+-) Camphor	0	0	0	0	0	0	0	0	0	0	0	0	+++	0	+	0	0	0	0	0	0	0	0	0	0	0	0	0	0	0	0	0	0	0	+++	++	+	0	0	0
Terpinolene	0	0	0	0	0	0	0	0	0	0	0	0	0	0	0	0	+++	++	++	++	+	++	0	0	0	0	0	0	0	0	0	0	0	0	0	0	0	0	0	0
Camphene	0	0	0	0	0	0	0	0	0	0	0	0	0	0	0	0	++	++	++	++	+	+++	0	0	0	0	0	0	0	0	0	0	0	0	0	0	0	0	0	0
Hexanal	0	0	0	0	0	0	0	0	0	0	0	0	0	0	0	0	0	0	0	0	0	0	+++	+++	0	+	0	0	0	0	+	0	0	0	0	0	0	0	0	0
1-Hexanol	0	0	0	0	0	0	0	0	0	0	0	0	0	0	0	0	0	0	0	0	0	0	+	+	+	+	0	0	0	0	0	0	0	0	0	0	0	0	0	0
Hexyl acetate	0	0	0	0	0	0	0	0	0	0	0	0	0	0	0	0	0	0	0	0	0	0	+	++	+	0	0	0	++	0	0	0	0	0	0	0	0	0	0	0
*E*2-Hexen-1-al	0	0	0	0	0	0	0	0	0	0	0	0	0	0	0	0	0	0	0	0	0	0	+	++	+	+	0	0	0	0	0	0	0	0	0	0	0	0	0	0
*Z*3-Hexenol	0	0	0	0	0	0	0	0	0	0	0	0	0	0	0	0	0	0	0	0	0	0	+++	0	+	0	++	++	0	0	++	0	0	0	0	0	0	0	0	0
*E*2-Hexen-1-ol	0	0	0	0	0	0	0	0	0	0	0	0	0	0	0	0	0	0	0	0	0	0	+	++	++	0	+++	+	0	0	+++	0	0	0	0	0	0	0	0	0
Total	15		10		2		5		3		1		4		3		5		9		2		4		2		2		1		2		3		7		4		2	

Number of insects = 86; spike frequencies: (+) 3–14 spikes/s, (++) 15–24 spikes/s, and (+++) >25 spikes/s; spike amplitudes: a low spike, b high spike.

## Data Availability

The datasets generated and analyzed during this study are available from the corresponding author on reasonable request.

## Supplementary Materials

The following supporting information can be downloaded at: https://www.mdpi.com/article/10.3390/insects14070653/s1, Figure S1: Comparison of responses between dosage in low and high spike amplitude neurons housed in trichoid sensilla in *Choristoneura fumiferana* grouped by female sex pheromone components in males (a) and females (b). Bars represented by different letters indicate significant differences between dosage (*p* < 0.05). Figure S2: Comparison of responses between female sex pheromone components in low and high spike amplitude neurons housed in trichoid sensilla in *Choristoneura fumiferana* grouped by dosage in males (a) and females (b). Bars represented by different letters indicate significant differences between female sex pheromone components (*p* < 0.05). Figure S3: Comparison of responses between low and high spike amplitude neurons housed in trichoid sensilla in *Choristoneura fumiferana* grouped by female sex pheromone component and a dosage in males and females. Bars represented by asterisks indicate significant differences between low and high spike amplitudes (*p* < 0.05). Figure S4: Comparison of responses between dosage in low and high spike amplitude neurons housed in trichoid sensilla in *Choristoneura fumiferana* grouped by host plant volatiles in males (a) and females (b). Bars represented by different letters indicate significant differences between dosages (*p* < 0.05). Figure S5: Comparison of responses between host plant volatiles in low and high spike amplitude neurons housed in trichoid sensilla in *Choristoneura fumiferana* grouped by dosage in males (a) and females (b). Bars represented by different letters indicate significant differences between female sex pheromone components (*p* < 0.05). Figure S6: Comparison of responses between low and high spike amplitude neurons housed in trichoid sensilla in *Choristoneura fumiferana* grouped by host plant volatile and a dosage in males and females. Bars represented by asterisks indicate significant differences between low and high spike amplitudes (*p* < 0.05). Figure S7: Comparison of responses between dosage in low and high amplitude spiking neurons housed in trichoid sensilla in *Choristoneura fumiferana* grouped by host plant volatiles in males. Bars represented by different letters indicate significant differences between dosage (*p* < 0.05). Figure S8: Comparison of responses between host plant volatiles in low and high spike amplitude neurons housed in trichoid sensilla in *Choristoneura fumiferana* within a dosage in males. Bars represented by different letters indicate significant differences between female sex pheromone components (*p* < 0.05). Figure S9: Comparison of responses between low and high spike amplitude neurons housed in trichoid sensilla in *Choristoneura fumiferana* within a host plant volatile and a dosage in males. Bars represented by asterisks indicate significant differences between low and high spike amplitudes (*p* < 0.05).
