# Supplementary material for: Pheromone and Host Plant Odor Detection in Eastern Spruce Budworm, Choristoneura fumiferana Clemens (Lepidoptera: Tortricidae)
Source: Insects. 2023 Jul 21;14(7):653. doi: 10.3390/insects14070653 (PMC10380843; doi:10.3390/insects14070653)
Supplement: Supplementary file 1 [file insects-14-00653-s001.zip › insects-2404397-supplementary.pdf]

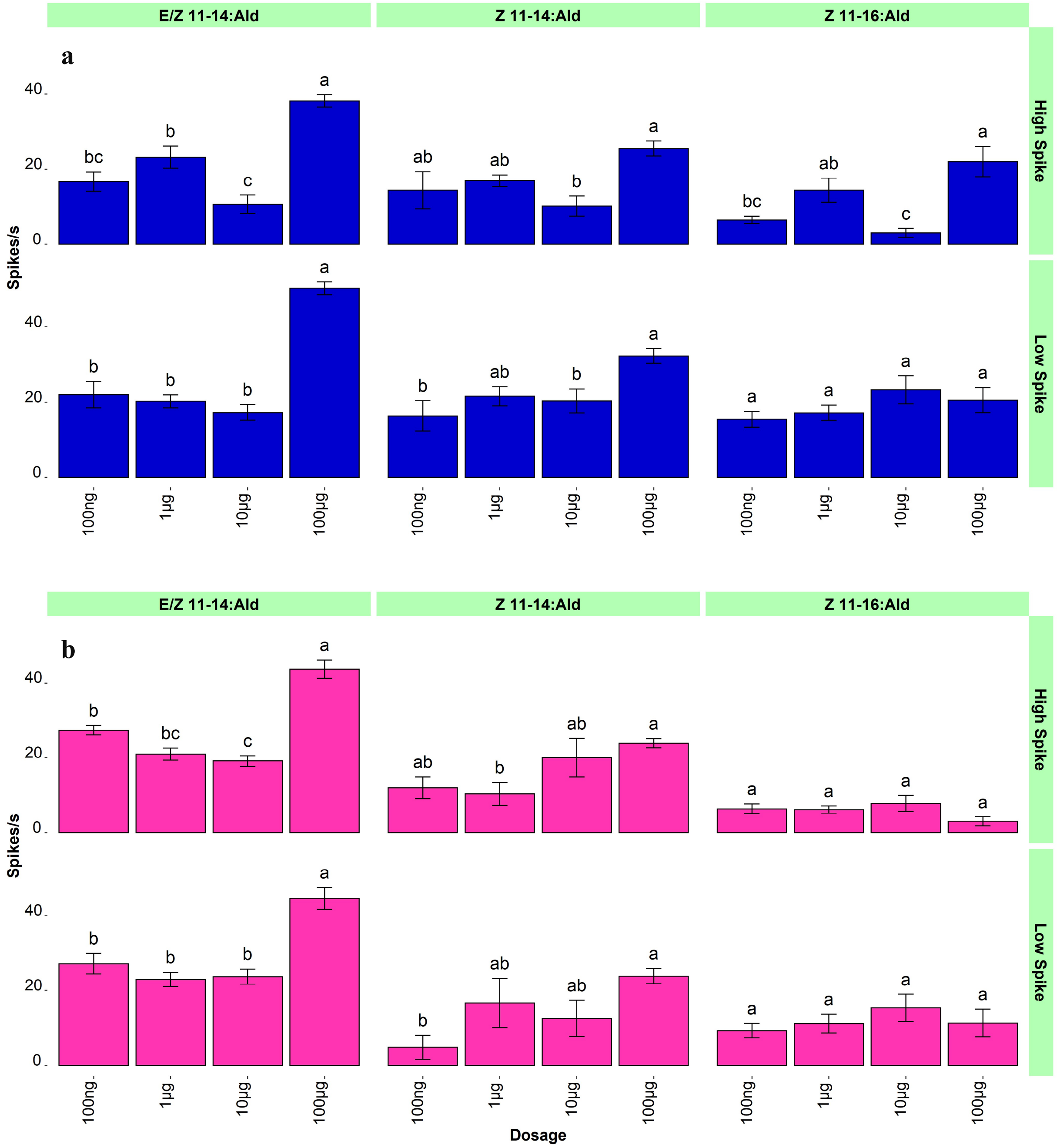

Figure S1. Comparison of responses between dosage in low and high amplitude spiking neurons housed in trichoid sensilla in *Choristoneura fumiferana* grouped by female sex pheromone components in males (a) and females (b). Bars represented by different letters indicate significant differences between dosage ($P < 0.05$).

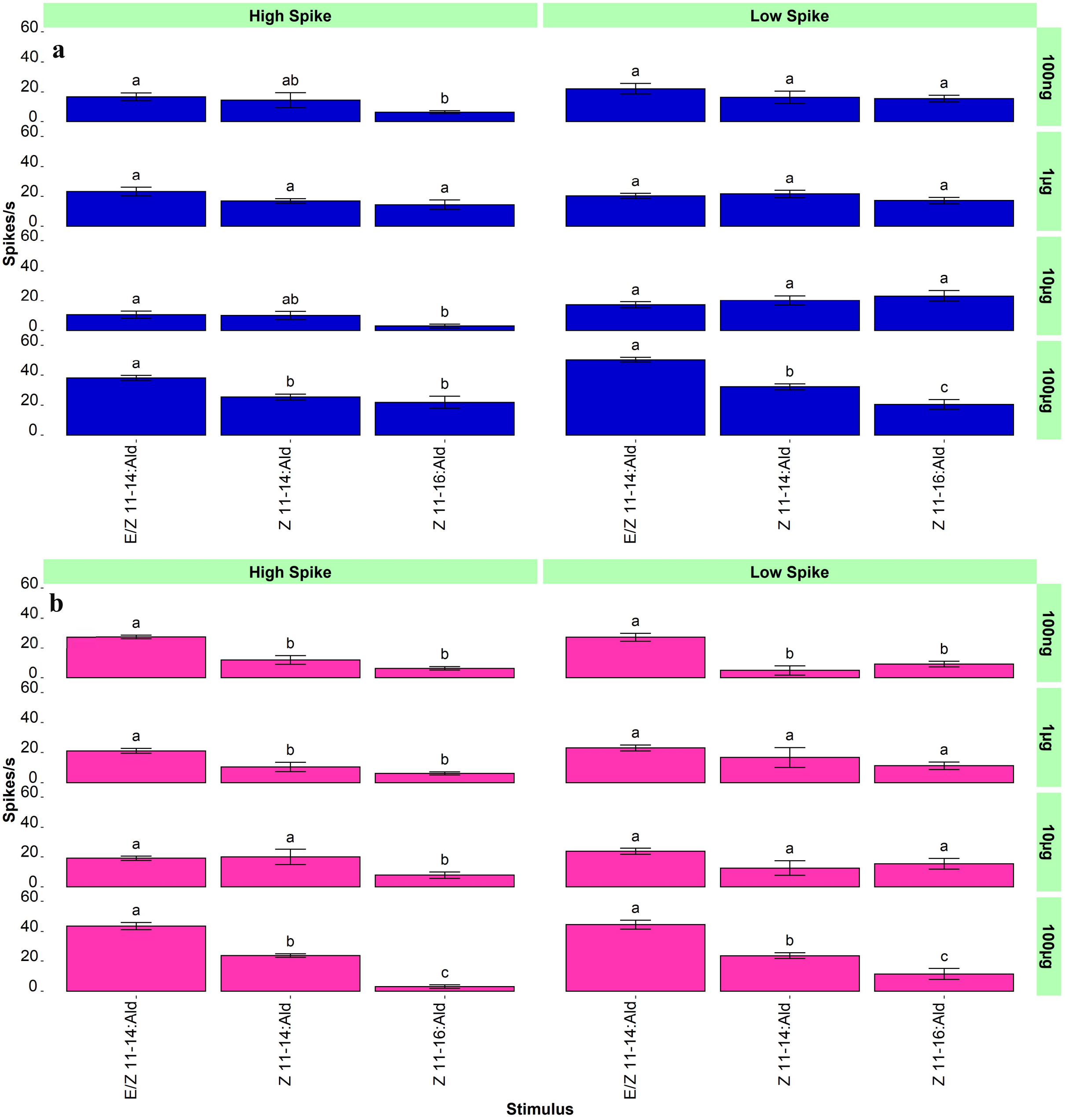

Figure S2. Comparison of responses between female sex pheromone components in low and high amplitude spiking neurons housed in trichoid sensilla in *Choristoneura fumiferana* grouped by dosage in males (a) and females (b). Bars represented by different letters indicate significant differences between female sex pheromone components ($P < 0.05$).

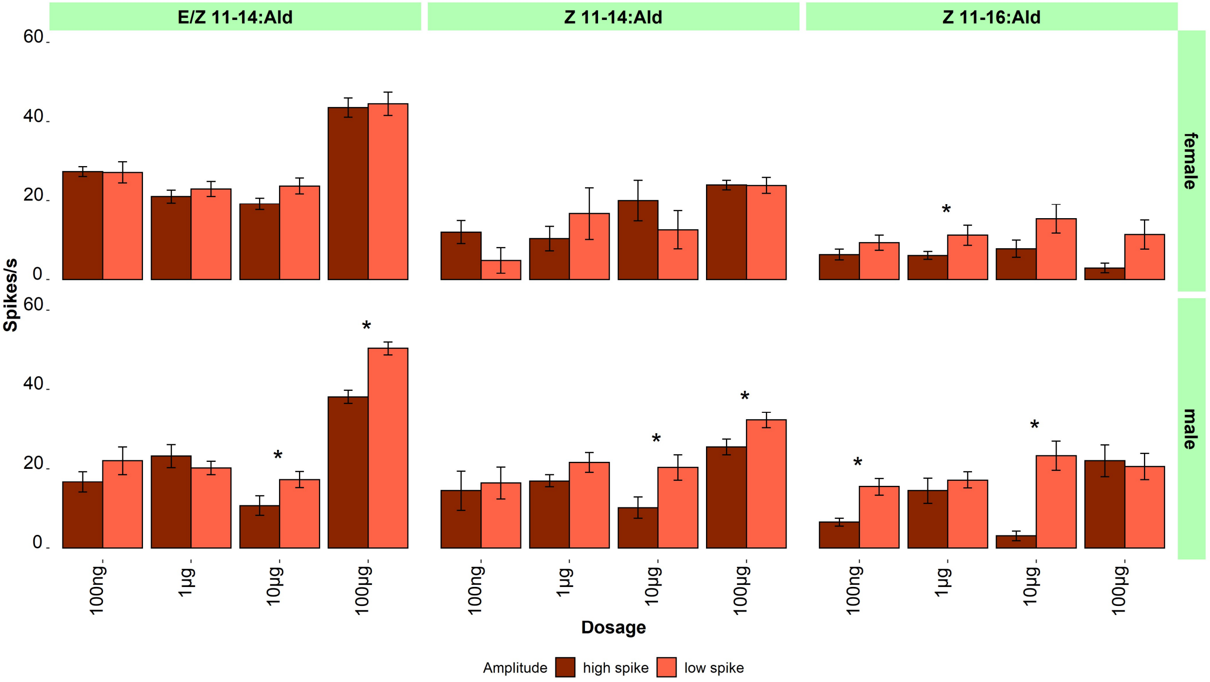

Figure S3. Comparison of responses between low and high amplitude spiking neurons housed in trichoid sensilla in *Choristoneura fumiferana* grouped by female sex pheromone component and a dosage in males and females. Bars represented by asterisks indicate significant differences between low and high spike amplitudes ($P < 0.05$).

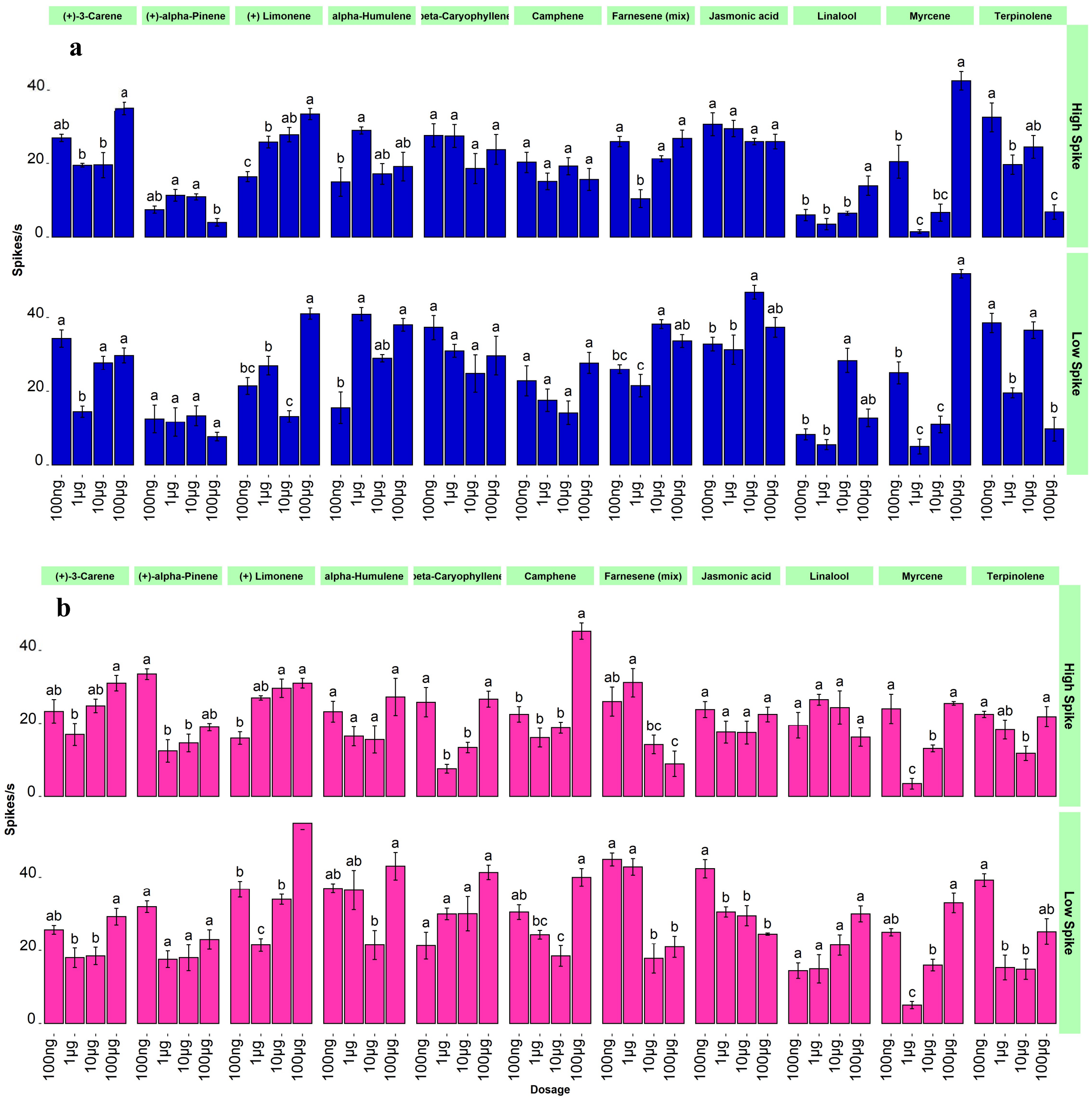

Figure S4. Comparison of responses between dosage in low and high amplitude spiking neurons housed in trichoid sensilla in *Choristoneura fumiferana* grouped by host plant volatiles in males (a) and females (b). Bars represented by different letters indicate significant differences between dosages (P < 0.05).

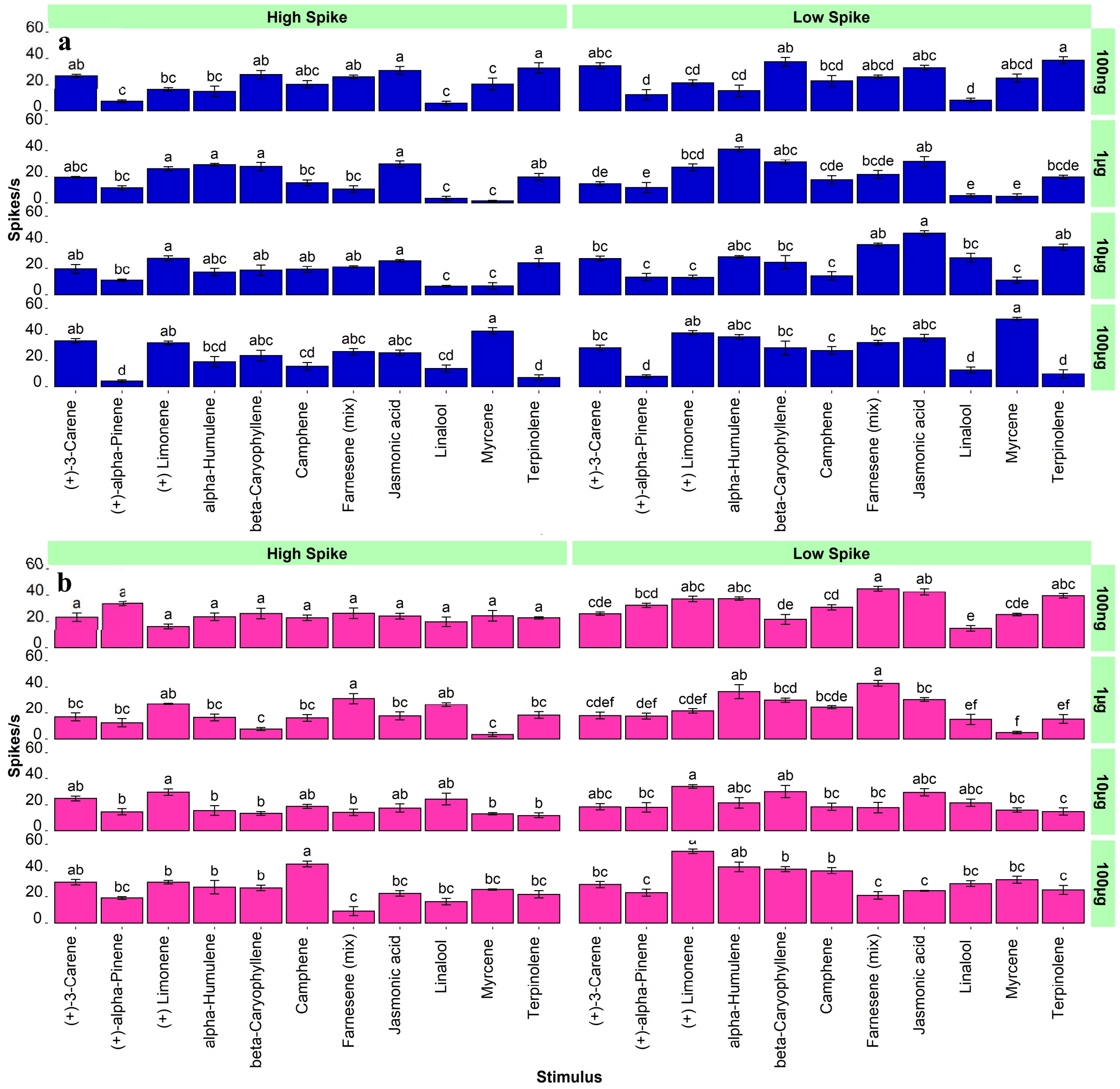

Figure S5. Comparison of responses between host plant volatiles in low and high amplitude spiking neurons housed in trichoid sensilla in *Choristoneura fumiferana* grouped by dosage in males (a) and females (b). Bars represented by different letters indicate significant differences between female sex pheromone components ($P < 0.05$).

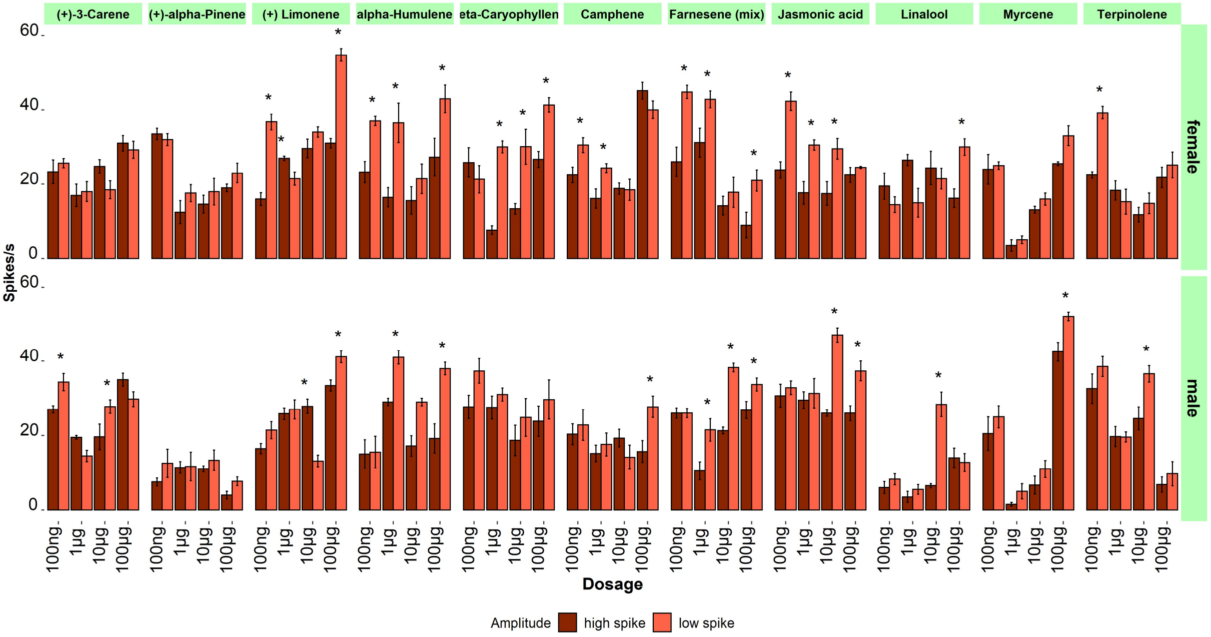

Figure S6. Comparison of responses between low and high amplitude spiking neurons housed in trichoid sensilla in *Choristoneura fumiferana* grouped by host plant volatile and a dosage in males and females. Bars represented by asterisks indicate significant differences between low and high spike amplitudes (P < 0.05).

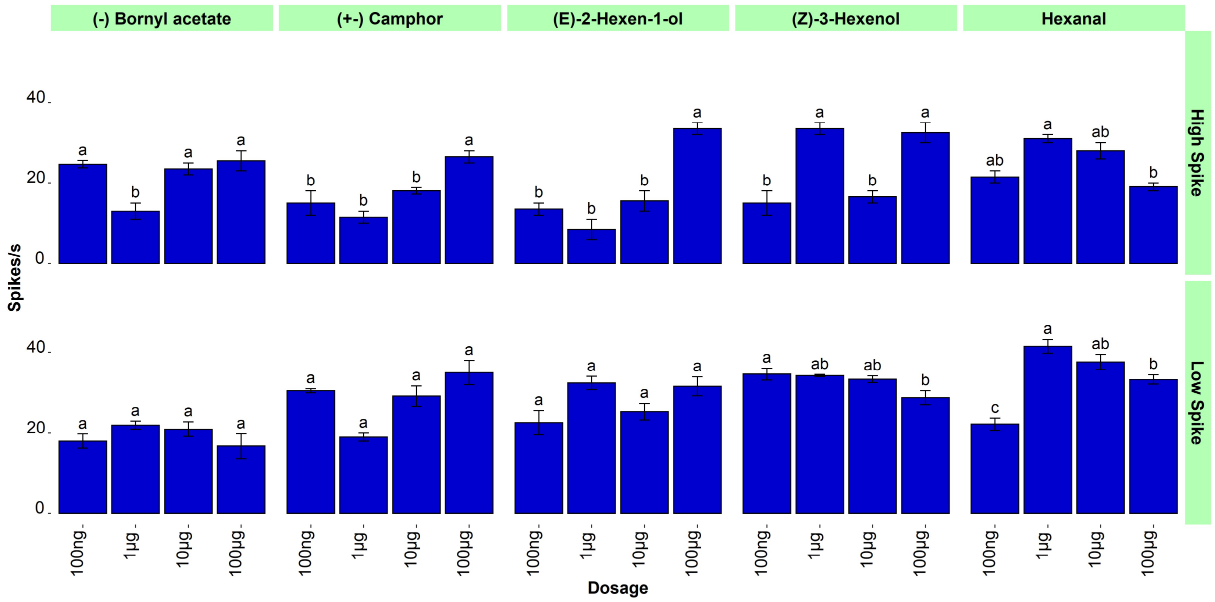

Figure S7. Comparison of responses between dosage in low and high amplitude spiking neurons housed in trichoid sensilla in *Choristoneura fumiferana* grouped by host plant volatiles in males. Bars represented by different letters indicate significant differences between dosage (P < 0.05).

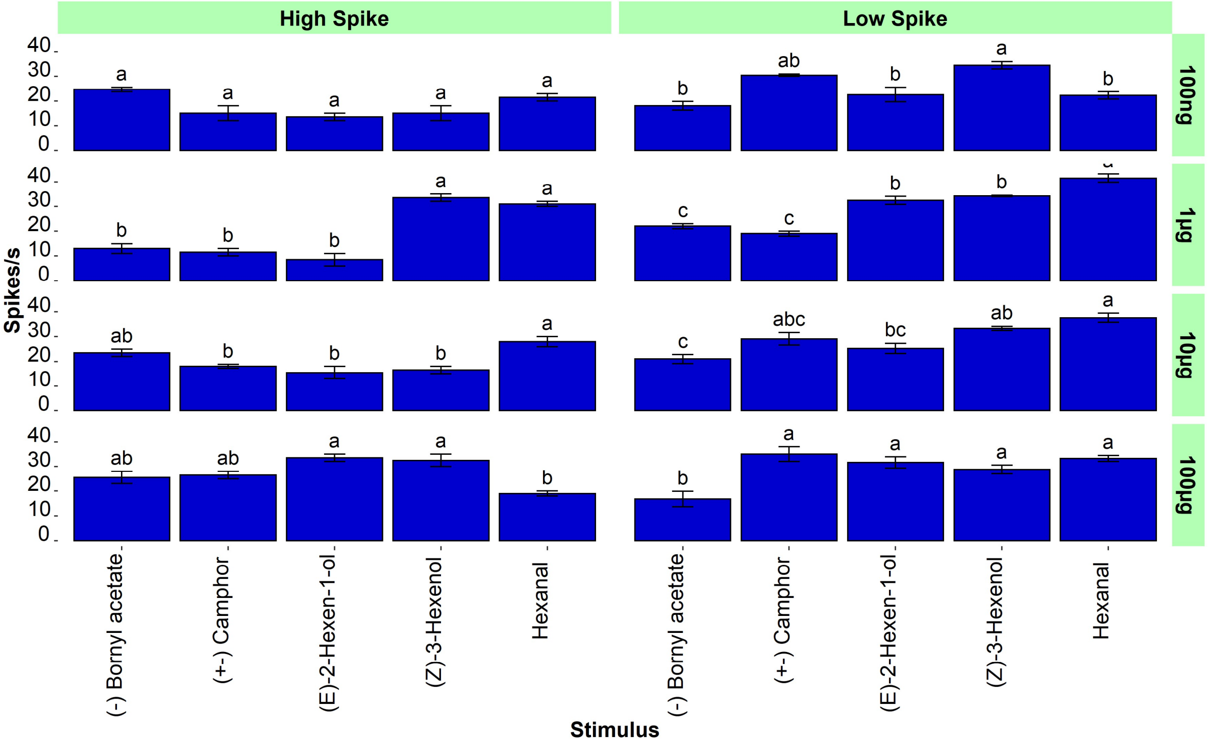

Figure S8. Comparison of responses between host plant volatiles in low and high amplitude spiking neurons housed in trichoid sensilla in *Choristoneura fumiferana* within a dosage in males. Bars represented by different letters indicate significant differences between female sex pheromone components ($P < 0.05$).

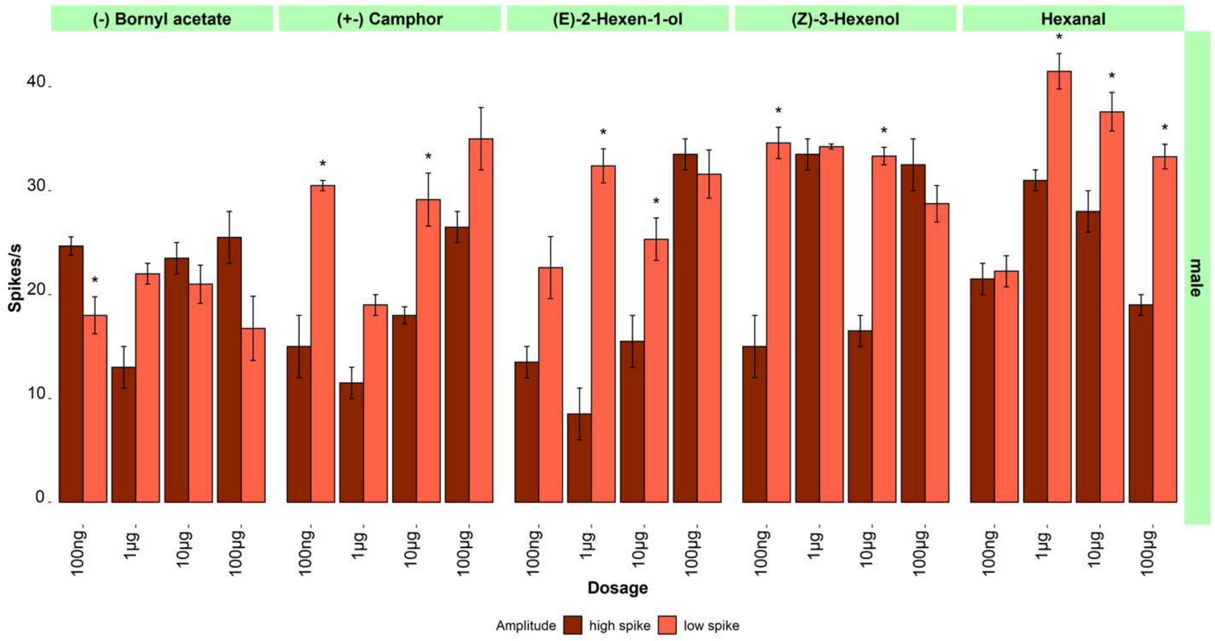

Figure S9. Comparison of responses between low and high amplitude spiking neurons housed in trichoid sensilla in *Choristoneura fumiferana* within a host plant volatile and a dosage in males. Bars represented by asterisks indicate significant differences between low and high spike amplitudes ($P < 0.05$).
